# Lack of expression of ALK and CD30 in breast carcinoma by immunohistochemistry irrespective of tumor characteristics

**DOI:** 10.1097/MD.0000000000016702

**Published:** 2019-08-09

**Authors:** Samer Nassif, Ziad M. El-Zaatari, Michel Attieh, Maya Hijazi, Najla Fakhreddin, Tarek Aridi, Fouad Boulos

**Affiliations:** Department of Pathology and Laboratory Medicine, American University of Beirut Medical Center, Beirut, Lebanon.

**Keywords:** ALK, breast cancer, carcinoma, CD30, middle east

## Abstract

CD30 is a member of the tumor necrosis factor family of cell surface receptors normally expressed in lymphocytes, as well as some lymphomas, but has been described in other malignancies. Anaplastic lymphoma kinase (ALK) is a tyrosine kinase receptor that belongs to the insulin receptor superfamily, and is normally expressed in neural cells, but has been detected in several malignancies. There is conflicting data in the literature that describes the expression of these receptors in breast cancer, and the aim of this study is to test the expression of CD30 and ALK in a cohort of Middle Eastern patients with breast carcinoma.

Cases of invasive breast cancer from the archives of AUBMC were reviewed over a period of 9 years, and the blocks that were used for immunohistochemical staining for ER, PR, Her-2/neu were selected. Immunohistochemical staining for CD30 (JCM182) and ALK (5A4 and D5F3) was performed.

Two hundred eighty-four cases were identified (2 cases were male), with a mean age of 55 ± 12. CD30 and ALK expression was not seen in any of the cases.

Our cohort showed complete negativity to both CD30 and ALK, adding to the conflicting data available in the literature, and more studies are needed to reliably identify a trend of expression of CD30 and ALK in breast carcinoma, especially in the Middle East.

## 1 Introduction

CD30, also known as Ki-1 or Ber-H2, is a member of the tumor necrosis factor family of cell surface receptors and is a known activation antigen in lymphocytes, being rarely expressed in non-lymphoid non-neoplastic cells.^[1,2]^ Its expression has been well studied in multiple lymphoid neoplasms including Hodgkin lymphoma,^[3,4]^ anaplastic large cell lymphoma,^[5,6]^ and diffuse large B-cell lymphoma,^[7]^ among others. However, CD30 expression has rarely been described in non-lymphoid tissues,^[1,8]^ and few studies have examined its expression in non-lymphoid neoplasms, namely epithelial tumors,^[9–13]^ sometimes with conflicting results.^[1,9,11]^ Specifically, very little data exists about CD30 expression in breast carcinoma or about its association with tumor characteristics. One study demonstrated CD30 protein expression by immunohistochemistry (IHC) in around 5% of triple negative breast cancer cases,^[1]^ and another recent study showed that CD30 protein expression by immunohistochemistry was seen with a higher sensitivity in breast cancer with a high CD30 gene RNA level.^[14]^ Such information could potentially be of great clinical benefit, especially given that anti-CD30 targeted therapy has been shown to be effective against CD30-positive neoplasms.^[15]^

Anaplastic lymphoma kinase (ALK, CD246) is a tyrosine kinase receptor that belongs to the insulin receptor superfamily. ALK is normally detected in neural cells and plays an important part in the early development of the nervous system.^[16]^ Although it is not found in normal non-neural adult tissues, its expression has been variably described in several malignancies including lymphoid tumors such as ALK-positive anaplastic large cell lymphoma,^[17,18]^ epithelial tumors such as lung adenocarcinomas,^[19]^ pancreatic ductal adenocarcinomas and neuroendocrine tumors,^[20]^ renal cell carcinomas,^[21]^ mesenchymal tumors such rhabdomyosarcoma,^[22]^ inflammatory myofibroblastic tumors,^[23]^ and neuroblastoma.^[24]^ In breast cancer, however, ALK expression and function are poorly understood, with relatively few studies describing ALK positive inflammatory^[25]^ and triple negative breast carcinomas.^[26]^ Similar to CD30 expression, knowledge of ALK expression in malignant breast tumors could be of value given that ALK-targeted therapy has been shown to be effective against certain neoplasms such as ALK expressing lung adenocarcinoma^[27]^ and neuroblastoma.^[28]^

To this date and to the best of our knowledge, there have been no studies examining the expression of both CD30 and ALK (2 different clones) in breast carcinoma, and their association, if any, with specific tumor characteristics. This is especially relevant in our country where almost half of breast cancer cases are diagnosed before age 50, and around 20% before age 40.^[29]^ Moreover, multiple studies have shown that breast cancer in young women tends to be more aggressive, with higher proportions of aggressive molecular groups, especially the Basal-Like/Triple Negative (BL/TN) subtype.^[30,31]^ It is therefore important to study the expression of ALK and CD30 in breast cancer in our population, particularly with the putative relationship between ALK expression and the BL/TN phenotype.^[32]^

## 2 Material and methods

This study was approved by the Institutional Review Board along with waiver of consent due to patient anonymity.

### 2.1 Case identification and block selection

Cases of invasive breast carcinoma were retrieved from the archives of the department of Pathology and Laboratory Medicine at the American University of Beirut Medical Center using the Laboratory Information system search engine, over a period of 9 years. The same blocks that were used for immunohistochemical staining for Estrogen and Progesterone Receptors and Her-2/neu expression were identified and selected for this study. Additionally, data was retrieved from the pathology reports including patient gender, age, tumor size, tumor type and grade, lymphovascular invasion, status of Estrogen Receptors, Progesterone Receptors, and Her-2/neu expression. All cases with missing information or unavailable paraffin blocks were excluded. All selected cases were internal (no referred blocks) and therefore fixed in 10% buffered formalin for an appropriate duration (6–48 hours).

### 2.2 Immunohistochemistry

Immunohistochemical staining for CD30 (JCM182), ALK (5A4) and ALK (D5F3) was performed using the Ventana immunostainer and following manufacturer-specified protocols. With each staining run, positive control tissues were used to ensure adequate staining performance.

## 3 Results

**Figure 1:**
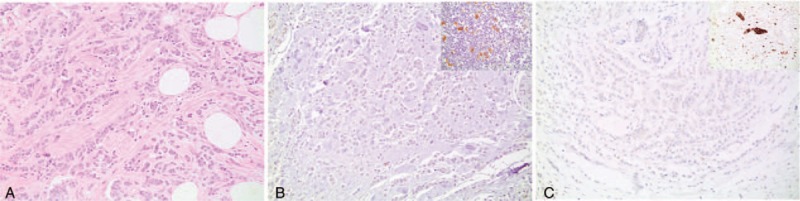
(A) Invasive carcinoma, hematoxilin and eosin, 400×. (B) Absence of CD30 staining in tumor cells, 400× (inlet: positive control stain). (C) Absence of ALK (D5F3) staining in tumor cells, 400× (inlet: positive control stain). The second ALK clone (5A4) also showed negative staining in tumor cells (not shown).

A total of 284 cases were identified, of which 282 were female and 2 were male. Patient age averaged 55 years with a standard deviation of 12 years. Tables 1 and 2 give an overview of the overall population studied, which spanned different tumor histologic and molecular types and grades, and included examples of cases with different tumor histologic characteristics and biomarker properties. CD30 and ALK expression was not seen in any of the examined cases, and therefore, no relationship between expression of these 2 markers and tumor characteristics could be established. Tissues in all the blocks that were used were adequately immunoreactive as was shown by the presence of positive internal controls when previously stained for estrogen and progesterone receptors. Additionally, control stains performed with each immunostaining run showed appropriate reactivity. Figure 1 illustrates the above findings.

**Table 1:**
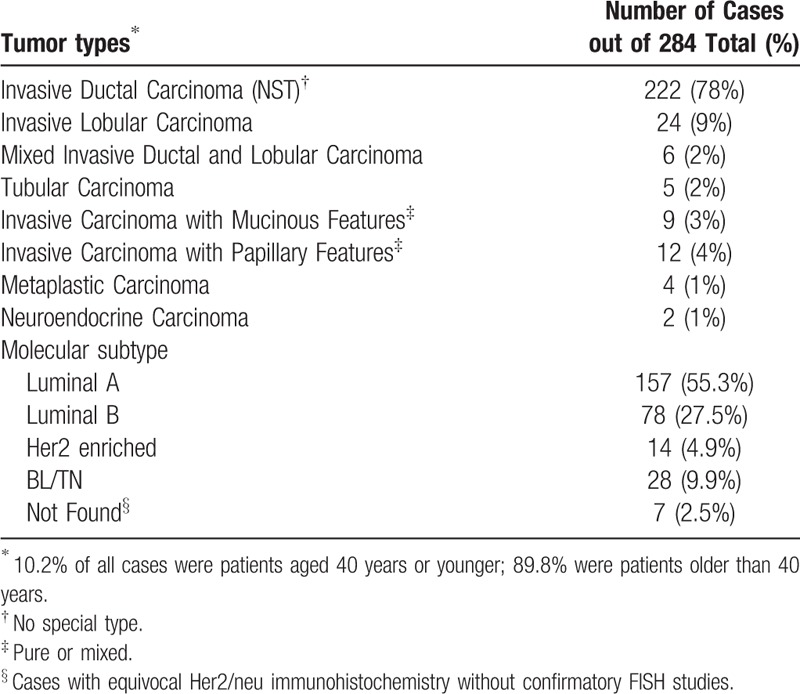
Summary of tumor morphologic types and molecular subtypes.

**Table 2:**
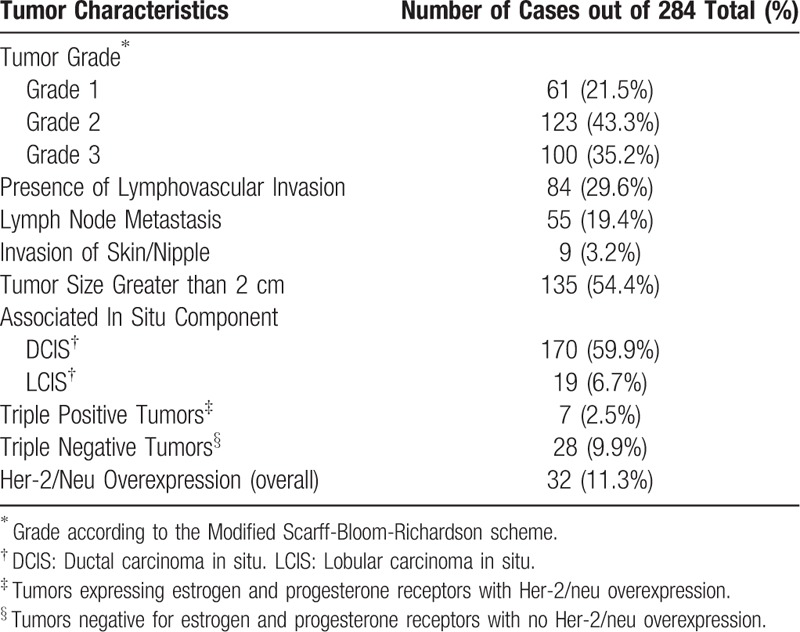
Summary of tumor characteristics.

## 4 Discussion

Confirming ALK positivity in breast carcinoma, be it through molecular or immunohistochemical techniques, is important as it may become a contributor to clinical decision making, given the known therapeutic effect of ALK inhibitors in ALK-positive lung adenocarcinoma and neuroblastoma.^[27,28]^ The relationship between ALK mutations or expression and breast carcinoma is however not fully understood, and various studies have shown different results, albeit with different methodologies. For example, while Lefebvre et al showed ALK mutations in a subset of hormone-positive metastatic breast cancers,^[33]^ Siraj et al showed a strong association between ALK expression and gene amplification (36% and 13%, respectively) and high-grade, triple negative, high proliferation index ductal carcinomas.^[32]^ Additionally, Perez-Pinera et al showed high-level nuclear and cytoplasmic ALK expression in various histologic types of breast carcinoma.^[34]^ Other studies found that ALK gene aberrations could be due to a copy number increase of either chromosome 2 as a whole,^[35]^ or the ALK gene specifically.^[36]^ Interestingly, according to Kim et al's study, copy number gain was associated with inflammatory breast cancer, but no significant correlation with positive immunohistochemical staining was found.^[36]^ Another example of discrepancy regarding ALK in breast cancers refers to the specific ALK-EML4 gene fusion typically identified in lung adenocarcinoma; this mutation was not detected in breast cancer by Fukuyoshi et al^[37]^ but detected in 2.4% of breast cancer cases by Lin et al.^[38]^

In addition to correlating very well with FISH negative and positive results, dichotomous immunohistochemical reactivity (0 vs 3+) with the ALK antibody seems to represent the strongest predictor of response to ALK inhibitor therapy,^[39]^ In fact, FISH-negative IHC-positive non-small cell lung cancer was repeatedly shown to respond to ALK-inhibitor therapy, while FISH-positive IHC-negative tumors failed treatment in 100% of cases.^[40–43]^

Therefore, we aimed in our study to assess ALK expression by immunohistochemistry in different types of breast cancer using 2 different ALK antibody clones. Despite the average size of our study sample, the selected cases adequately represent the broad spectrum of breast cancer presentations at our institution. Results were similar with both clones in that there was complete absence of ALK expression in all stained samples.

Our results contrast with previous studies where ALK expression in breast cancer was demonstrated immunohistochemically^[34]^ and even when using the same antibody clone.^[32]^ The reasons for this discrepancy are not evident, but could be related to population-dependent genetic differences. In view of the marked variation in results in ALK expression/mutation in breast cancer, questions arise whether ALK-targeted treatment strategies in breast carcinoma can be of benefit.

Our study also showed complete lack of expression of CD30 in the selected breast cancer cases. These results argue against testing for CD30 positivity in breast cancer, and cast significant doubt on the potential for anti-CD30 targeted therapy in advanced or refractory breast cancer cases.

Of note, one limitation to this study was the lack of testing for ALK and CD30 mutations by molecular techniques due to funding restrictions. This could be the subject of future projects.

In summary, we showed the lack of ALK and CD30 immunoreactivity in breast cancer in our cohort, irrespective of tumor characteristics. The conflicting results between our study and that of Siraj et al warrant further investigation, given that 36% of their cases were IHC positive for ALK, whereas our cohort showed complete negativity using 2 different antibody clones. This raises the suspicion of possible methodological contributors from either study to this significant difference between 2 somewhat comparable patient populations. We, therefore, believe that studies on the expression of both ALK and CD30 in breast carcinoma are still necessary, given the potential benefit of targeted therapy on breast cancers with aggressive biology and poor response to conventional chemotherapeutic regimens.

## Author contributions

**Conceptualization:** Samer Nassif, Ziad M. El-Zaatari, Maya Hijazi, Najla Fakhreddin, Fouad Boulos.

**Data curation:** Samer Nassif, Ziad M. El-Zaatari, Michel Attieh, Maya Hijazi, Tarek Aridi, Najla Fakhreddin, Fouad Boulos.

**Formal analysis:** Samer Nassif, Ziad M. El-Zaatari, Tarek Aridi, Fouad Boulos.

**Funding acquisition:** Samer Nassif, Fouad Boulos.

**Investigation:** Samer Nassif, Michel Attieh, Maya Hijazi, Najla Fakhreddin, Fouad Boulos.

**Methodology:** Samer Nassif, Ziad M. El-Zaatari, Maya Hijazi, Tarek Aridi, Najla Fakhreddin, Fouad Boulos.

**Project administration:** Samer Nassif, Fouad Boulos.

**Resources:** Samer Nassif, Fouad Boulos.

**Software:** Samer Nassif, Fouad Boulos.

**Supervision:** Samer Nassif, Najla Fakhreddin, Fouad Boulos.

**Validation:** Samer Nassif, Fouad Boulos.

**Visualization:** Samer Nassif, Michel Attieh, Maya Hijazi, Tarek Aridi, Fouad Boulos.

**Writing – original draft:** Samer Nassif, Ziad M. El-Zaatari, Michel Attieh, Maya Hijazi, Tarek Aridi, Najla Fakhreddin, Fouad Boulos.

**Writing – review & editing:** Samer Nassif, Ziad M. El-Zaatari, Michel Attieh, Fouad Boulos.
